# Dynamic changes of phenotypically different circulating tumor cells sub-populations in patients with recurrent/refractory small cell lung cancer treated with pazopanib

**DOI:** 10.1038/s41598-018-20502-1

**Published:** 2018-02-02

**Authors:** Ippokratis Messaritakis, Eleni Politaki, Fillipos Koinis, Dimitris Stoltidis, Stella Apostolaki, Maria Plataki, Eleftheria-Kleio Dermitzaki, Vassilis Georgoulias, Athanasios Kotsakis

**Affiliations:** 1 Laboratory of Tumor Cell Biology, Medical School, University of Crete, Heraklion, Crete, Greece; 2 Department of Medical Oncology, University General Hospital of Heraklion, Crete, Greece

## Abstract

The aim of the study was to investigate the effect of 2^nd^-line pazopanib on the different CTCs subpopulations in SCLC patients and evaluate the clinical relevance of their changes. Different CTCs subpopulations were evaluated before pazopanib initiation (n = 56 patients), after one-cycle (n = 35) and on disease progression (n = 45) by CellSearch and double immunofluorescence using anti-CKs and anti-Ki67, anti-M30 or anti-Vimentin antibodies. Before treatment, CTCs were detected in 50% of patients by CellSearch whereas 53.4%, 15.5% and 74.1% patients had CK^+^/Ki67^+^, CK^+^/M30^+^ and CK^+^/Vim^+^ CTCs, respectively. One pazopanib cycle significantly decreased the number of CTCs as detected by CellSearch (*p* = 0.043) as well as the number of CK^+^/Ki67^+^ (*p* < 0.001), CK^+^/M30^+^ (*p* = 0^.^015) and CK^+^/Vim^+^ (*p* < 0^.^001) cells. On disease progression, both the incidence and CTC numbers were significantly increased (CellSearch, *p* = 0.027; CK^+^/Ki67^+^, *p* < 0.001; CK^+/^M30^+^, *p* = 0.001 and CK^+^/Vim^+^, *p* < 0^.^001)^.^ In multivariate analysis, the detection of CK^+^/Vim^+^ CTCs after one treatment cycle (HR: 7.9, 95% CI: 2.9–21.8; *p* < 0.001) and CTCs number on disease progression, as assessed by CellSearch, (HR: 2.0, 95% CI: 1.0–6.0; *p* = 0.005) were emerged as independent factors associated with decreased OS. In conclusion, pazopanib can eliminate different CTC subpopulations in patients with relapsed SCLC. The analysis of CTCs could be used as a dynamic biomarker of treatment efficacy.

## Introduction

Pazopanib is a tyrosine multikinase inhibitor, targeting the VEGFR-1, VEGFR-2, VEGFR-3, PDGF and c-KIT^1^. In preclinical models, pazopanib inhibited phosphorylation of VEGFR-2, c-KIT and PDGFR-β receptors in a dose-dependent manner^2^. Pazopanib has already demonstrated substantial efficacy in renal cell cancer and sarcomas^3,4^.

SCLC accounts for about 13% of all lung cancer cases in the USA and at the time of presentation, about two thirds of the patients present extensive-stage disease^5^. Despite its chemo- and radio-sensitivity, the prognosis of the disease is poor since the early response is almost always followed by recurrent or metastatic disease and patients die from disease progression^6–8^. SCLC spreads through the hematogenous route since it has been demonstrated that the density of microvessels and the expression of vascular endothelial growth factor (VEGF) is directly related to the development of metastases and poor prognosis^9,10^. Thus, targeting angiogenesis with pazopanib in patients with SCLC is an attractive choice due to the limited treatment options. Moreover, the high risk of disseminated disease highlights the need for alternative treatments that act directly on the growth and invasiveness of SCLC.

Detection of circulating tumor cells (CTCs) has been shown to be associated with disease prognosis and can serve for the monitoring of treatment efficacy in different cancer types, including SCLC^3,4,11–15^. Moreover, several studies have shown that there is a substantial phenotypic and molecular heterogeneity of CTCs in different tumor types^14,16,17^. Our group has previously reported that CTCs from patients with early and metastatic breast cancer may have either a proliferating (CK^+^/Ki67^+^) or an apoptotic (CK^+^/M30^+^) as well as an Epithelial-to-Mesenchymal Transition (EMT; CK^+^/Vim^+^ or CK^+^/TWIST^+^) phenotype^18,19^. In SCLC, Hou *et al*.^13^ reported the presence of apoptotic CTCs in a substantial proportion of patients while the CTC number was correlated with the patients’ outcome; in addition, the molecular characterization of CTCs revealed the presence of solitary Ki67^+^ and bcl-2^+^ CTCs. It is interesting to note that both apoptotic (CK^+^/M30^+^) and proliferating (CK^+^/Ki67^+^) CTCs were never observed in circulating tumor microemboli (CTM) in contrast to bcl-2^+^ CTCs which could be detected in CTMs^13^. These data clearly suggest that the different subpopulations of CTCs in patients with SCLC may have a differential biologic behavior which could characterize the aggressive potential of the disease and its resistance to treatment^20,21^. Furthermore, it has been shown that CTCs from patients with early and metastatic breast cancer express VEGF and VEGFR2^22^, suggesting the existence of an autocrine mechanism which could be involved in the metastatic process.

Based on these data, a correlative translational research study was undertaken in order to investigate the effect of 2nd line pazopanib on the different subpopulations of CTCs in patients with SCLC and, more especially, to evaluate the proliferative, apoptotic and EMT status of these cells.

## Patients and Methods

### Patients’ eligibility criteria

Patients were enrolled in an open-label, multicenter, phase II study, conducted by the Lung Cancer Working Group of the Hellenic Oncology Research Group (HORG) (*Clinicaltrialsgov*: NCT01713296 reg. date 27 Sept 2012) (*EudraCT number*: 2011-000879-14). Patients with platinum-sensitive (relapse >90 days after completion of front-line treatment) and -resistant/refractory SCLC (unresponsive to initial treatment and relapse <90 days following completion of front-line treatment) were enrolled in a multicentre phase II study. The primary end point was the progression-free survival rate at week 8 in each cohort. Inclusion criteria for enrolment are previously presented in detail^23^. In brief, patients with histologically or cytologically confirmed SCLC and radiologically documented progressive disease after first-line chemotherapy or chemoradiotherapy were recruited. Additional eligibility criteria included age ≥18 years, measurable disease according to RECIST v.1.1^24^, adequate liver, renal and bone marrow function, ability to swallow oral medications and an ECOG performance status of ≤2. Pazopanib was given orally, once daily. Toxicity was graded according to the Common Terminology Criteria for Adverse Events^25^. Tumor response assessment (by physical examination and CT scans) using the RECIST criteria v.1.1 was performed every two cycles (each cycle of 28 days), or earlier if clinically indicated. On evaluation, a Partial Response (PR) was considered when at least a 30% decrease in the sum of diameters of lesion, taking as reference the baseline sum diameters^24^. Progressive Disease (PD) was considered when at least a 20% increase in the sum of diameters of lesion, taking as reference the smallest sum on study^24^. Stable Disease (SD) was considered when neither sufficient shrinkage to qualify for PR nor sufficient increase to qualify for PD, taking as reference the smallest sum diameters while on study^24^. Moreover, as limited (LD) was considered a disease contained in one side of the chest and might have reached the lymph nodes of the same side. As extended (ED) was considered a disease that is widely spread to the lung, lymph nodes or other parts of the body^26^. Treatment was discontinued in case of unacceptable toxicity, treatment delay >2 weeks due to insufficient recovery from toxicity or if more than two dose reductions were required. Patients continued treatment until disease progression or occurrence of unacceptable toxicity. The main outcomes of the clinical trial were: (a) pazopanib showed a promising activity as second-line treatment in patients with platinum-sensitive SCLC, and (b) CTCs monitoring and enumaration could contribute to the early detection of the patients who most likely will benefit from pazopanib and might serve as an early reliable surrogate biomarker that predict for response according to radiographic criteria. The study has been approved by the National Drug Organization (EOF) and the National Ethics Committees (EED) as well as by the Scientific and Ethics Committees of the participating Institutions and all experiments were performed in accordance with relevant guidelines and regulations. All patients signed a written informed consent in order to participate in the study and had the right to withdraw consent.

### Patient samples and cytospin preparation

Peripheral blood (20 mL in EDTA and 7.5 ml in CellSearch Save preservative tubes; Veridex LLC, Raritan, NJ, USA) was obtained from patients before the administration of the 1^st^ cycle of pazopanib (n = 56 patients), after the administration of one cycle (n = 35 patients) and at the time of clinical or radiological disease progression (n = 45 patients). A CONSORT diagram demonstrating the flow of enrolled patients has been previously reported^27^. All blood samples were obtained at the middle-of-vein puncture after the first 5 mL of blood were discarded to avoid contamination with epithelial cells from the skin.

Peripheral blood mononuclear cells (PBMCs) were isolated by Ficoll–Hypaque density (*d* = 1, 077 g/mL; Sigma-Aldrich Chemie GmbH, Germany) centrifugation at 530 × g for 30 min at ambient temperature. Aliquots of 5 × 10^5^ PBMCs were cyto-centrifuged at 650 × g for 2 min on glass microscope slides. Cytospins were dried and stored at −80 °C until use. Two slides (10 × 10^5^ PBMCs) from each patient were analyzed at each time point.

### Detection of CTCs using the CellSearch platform

The semi-automated CellSearch assay (CS) was used for the enumeration of CTCs in peripheral blood. CTC morphology was confirmed in all cases and analysis was performed with the CellTracks Analyser II by two experienced biologists (EP and SA). Results are expressed as number of CTCs/7.5 ml blood. Patients with blood samples containing ≥5 CTCs/7.5 ml of blood were considered to be positive compared to patients with <5 CTCs/7.5 ml of blood who considered negative, according to previous evaluations of the CellSearch system both in SCLC^28–30^ and other neoplasms^12,31–33^.

### Double Immunofluorescence Assay

The presence of CTCs in PBMCs’ cytospins was investigated using monoclonal antibodies against Ki67 (a proliferation marker; Abcam, Cambridge, UK), M30 (an apoptosis marker; CytoDEATH fluorescein, Roche, Manheim, Germany) and Vimentin (an EMT marker; Santa Cruz, Santa Cruz, CA, USA). In addition, the epithelial origin of the cells was confirmed using the mouse A45-B/B3 antibody (detecting CK8, CK18 and CK19 and will be referred as CK^+^ in the text; Micromet, Munich, Germany). Moreover, cytospins from patients without detectable CTCs by CS were double stained with the anti-Ki67, anti-M30 or anti-Vimentin and the mouse anti-EpCAM (Acris Antibodies GmbH, Germany) antibodies. The cytomorphological criteria proposed by Meng *et al*. (i.e. high nuclear/cytoplasmic ratio, larger cells than white blood cells) were used to characterize a CK-positive cell as a CTC^34^. Double immunofluorescence staining was performed as described previously^22,35^. Briefly, fixed PBMCs cytospins were incubated with the appropriate primary and secondary antibodies for 60 mins. Ki67 and Vimentin were labelled with the anti-rabbit Alexa 555 (Molecular Probes, Invitrogen, Carlsbad, CA, USA), M30 was an already fluorescein-conjugated mouse antibody and CK was detected using the corresponding secondary fluorescein isothiocyanate (FITC) fluorochrome or the anti-mouse Alexa 555 (Molecular Probes). Finally, antifade reagent with 4′,6-diamidino-2-phenylindole (DAPI) (Molecular Probes) was added to each sample for cell nuclear staining. The omission of the first antibody (anti-Ki67, anti-M30, anti-Vim, anti-CK) has been used in negative control experiments. The sensitivity and the specificity of the staining with the different antibodies have been previously reported^14,19,22,35^. Slides were analyzed visually using a fluorescence microscope (Leica DM 2500, Heidelberg, Germany) by two experienced biologists (IM and SA) and the results are expressed as number of CTCs/10^6^ PBMCs.

### Cell Lines

The human SKBR3, MDA-MB231 and HeLa cell lines were obtained from the American Type Culture Collection (ATCC, Manassas, VA, USA) and used as positive controls. SKBR3 cells, treated in the presence or absence of staurosporine, were used as positive controls for CK/M30 expression^19,36^. Cyto-centrifuged MDA-MB231 cells were used as positive controls for CK/Ki67 expression. The HeLa adenocarcinoma cells were used as positive controls for CK/Vimentin expression. In addition, cells from the different cell lines were double stained with anti-CD45 (Common Leukocyte Antigen; Santa Cruz) and either anti-Ki67 or anti-M30 or anti-Vim antibodies in order to exclude possible ectopic expression on such cells.

SKBR3 cells were cultured in McCoy’s 5A GlutaMAX supplemented with 10% fetal bovine serum (FBS) (Gibco BRL Life Technologies, Rockville, MD, USA). MDA-MB231 cells were cultured in Dulbecco’s modified Eagle’s medium (DMEM) GlutaMAX supplemented with 10% FBS. MDA-MB231 cells were cultured in Dulbecco’s modified Eagle’s medium (DMEM) GlutaMAX supplemented with 10% FBS. HeLa cells were cultured in 1:1 (vol/vol) DMEM (Gibco-BRL) supplemented with 10% foetal bovine serum (FBS) (Gibco-BRL), 2 mmol L-glutamine (Gibco-BRL) and 50 mg/mL penicillin/streptomycin (Gibco-BRL). All cells were maintained in a humidified atmosphere of 5% CO_2_ in air. Sub-cultivation of all cell lines was performed using 0.25% trypsin and 5 Mmol ethylene-diamine-tetra-acetic acid (EDTA) (Gibco BRL). All experiments were performed during the logarithmic growth phase of each cell line.

### Study design and statistics

This phase II study had a Simon’s Mini-Max 2-stage design, requiring that 7 out of 19 patients achieving disease control at 8-Weeks during the first stage and 20 additional patients in the second stage. Progression-free survival (PFS) and overall survival (OS) were calculated from the day of enrolment to the first clinical or radiologic evidence of disease progression or death, respectively. The evaluation for the presence of positive cells was done blindly to clinical data. The potential association between baseline clinico-pathological characteristics and the detection of CTCs was compared using the 2-sided Fisher exact test for categorical variables. Coefficient correlation between variables was performed using the Spearman test. The association of risk factors with time-to-event endpoints was analyzed using the log rank test and, the Kaplan–Meier method was used to plot the corresponding PFS and OS curves. Univariate and multivariate Cox proportional hazards regression models with hazard ratios (HR) and 95% CIs were used to assess the association between potential prognostic factors and PFS or OS. Statistical significance was set at *p* = 0.05. All statistical analysis was performed using the SPSS v.20 software (IBM Corp., Armonk, NY, USA).

### Data availability

The datasets generated during and/or analyzed during the current study are available from the corresponding author on reasonable request.

## Results

### Patients’ characteristics

Fifty-eight patients were enrolled during the period 10/2011-4/2015 and baseline CTC analysis was performed in 56 of them. The patients’ characteristics are listed in Table 1. The patients’ median age was 66 years, 48 (82.8%) were men, 52 (89.7%) had a performance status (PS ECOG) 0–1, and 44 (75.9%) had extensive disease (ED); twenty-one (36.2%) patients had liver and/or brain metastases and 12 (20.7%) bone localizations; additionally, 23 (39.7%) patients had increased LDH serum levels and 38 (65.5%) anaemia. Thirty-seven (63.8%) and 21 (37.9%) patients had platinum-sensitive and platinum-resistant disease, respectively whereas 33 (56.9%) patients experienced an objective response (Partial response-PR) with front-line treatment.

**Table 1:** Patients demographics.

		**CellSearch**		
	**All patients (%) (N** = **58)**	≥**5 CTC (%)(N** = **28)**	<**5 CTCs (%)(N** = **28)**	***p*** **value**
Median Age	66 (range, 39–82)	65 (range, 46–79)	67 (range, 39–82)	
Gender				
Male	48 (82, 8%)	25 (44, 6)	21 (37, 5)	0, 148
Female	10 (17, 2%)	3 (5, 4)	7 (12, 5)	
PS (ECOG)				
0–1	52 (89, 7%)	25 (48, 1)	25 (48, 1)	0, 755
≥2	2 (3, 4%)	1 (1, 9)	1 (1, 9)	
Unknown	4 (6, 9%)	2 (3, 4)	2 (3, 4)	
Disease Extent				
Limited disease-LD	14 (24, 1%)	3 (5, 4)	10 (17, 9)	0, 028
Extensive disease-ED	44 (75, 9%)	26 (44, 6)	18 (32, 1)	
Lactate Dehydrogenase-LDH				
Normal	31 (53, 4%)	14 (26, 9)	15 (28, 8)	0, 548
High	23 (39, 7%)	12 (23, 1)	11 (21, 2)	
Unknown	4 (6, 9%)	2 (3, 4)	2 (3, 4)	
Hematocrit-Hct				
Normal	20 (34, 5%)	7 (12, 5)	11 (19, 6)	0, 196
Low	38 (65, 5%)	21 (37, 5)	17 (30, 4)	
Platelets-PLT				
Normal	52 (89, 7%)	25 (44, 6)	25 (44, 6)	0, 5
High	6 (10, 3%)	3 (5, 4)	3 (5, 4)	
Response to 1st Line				
Partial Response-PR	33 (56, 9%)	11 (19, 6)	20 (35, 7)	0, 02
Stable Disease-SD	21 (36, 2%)	13 (23, 2)	8 (14, 3)	
Disease progression -PD	4 (6, 9%)	4 (7, 1)	0 (0)	
Localization				
Liver/Brain	21 (36, 2%)	11 (19, 6)	9 (16, 1)	0, 227
Bones	12 (20, 7%)	9 (16, 1)	3 (5, 4)	
Unknown	25 (43, 1%)	8 (14, 3)	16 (28, 5)	
Platinum Resistance				
Sensitive	37 (63, 8%)	17 (30, 4%)	18 (32, 1%)	0, 500
Resistant	21 (36, 2%)	11 (19, 6%)	10 (17, 9%)	

### Detection of CTCs before the initiation of systemic treatment using the CS assay

Twenty-eight (50.0%) patients had increased numbers of CTCs at baseline (median: 71 CTCs/7.5 ml blood; range 5–11143 CTCs/7.5 ml) (Tables 1 and 2). Among the 28 (50.0%) patients with a low number of CTCs (range, 0–4 CTCs/7.5 ml of blood), 16 (57.1%) had ≥1 CTC/7.5 ml (Supplementary Table 1). The detection of an increased number of CTCs in patients was associated with ED and resistance to front-line treatment (*p* = 0.028 and *p* = 0.02, respectively) (Table 1).

**Table 2:** Detection of different sub-populations of CTCs during treatment with pazopanib.

	**Baseline**		**Post-1** ^**st**^ **cycle**		**Progression**	
	**Nb of +ve pts (%)**	**Median Nb of CTCs (range)**	**Nb of +ve pts (%)**	**Median Nb of CTCs (range)**	**Nb of +ve pts (%)**	**MedianNb of CTCs (range)**
CellSearch	28/56 (50, 0%)	71 (5–11143)	7/35 (20, 0%)*	12 (5–804)^‡^	18/45 (40, 0%)^a^	73 (5–16806)^†^
CK^+^/Ki67^+^	31/58 (53, 4%)	2 (0–19)	8/37 (21, 6%)**	0 (0–2)^‡‡^	40/48 (83, 3%)^b^	11 (0–79)^††^
CK^+^/Ki67^−^	39/58 (67, 2%)	2 (0–57)	12/37 (32, 4%)	0 (0–19)	37/48 (77, 1%)^c^	2 (0–46)^†††^
CK^+^/M30^+^	9/58 (15, 5%)	0 (0–7)	13/37 (35, 1%)***	0 (0–7)^‡‡‡^	5/48 (10, 4%)^d^	0 (0–24)^††††^
CK^+^/M30^−^	42/58 (72, 4%)	4 (0–68)	9 /37(24, 3%)****	0 (0–16)^‡‡‡‡^	38/48 (79, 2%)^e^	6 (0–84)^†††††^
CK^+^/Vim^+^	43/58 (74, 1%)	8 (0–196)	11/37 (29, 7%)*****	0 (0–21)^‡‡‡‡‡^	36/48 (75, 0%)^f^	11 (0–146)^††††††^
CK^+^/Vim^−^	33/58 (56, 9%)	1 (0–46)	9/37 (24, 3%)	0 (0–9)	28/48 (58, 3%)^g^	1 (0–24)^†††††††^

p value: Baseline vs Post 1st cycle: *0,043; **<0,001; ***0,015; ****<0,001; *****<0,001; ^‡^0,043; ^‡‡^<0,001; ^‡‡‡^0,015; ^‡‡‡‡^<0,001; ^‡‡‡‡‡^<0,001.

p value: Post 1st vs Progression: ^a^0,027; ^b^<0,001; ^c^0,004; ^d^0,001; ^e^<0,001; ^f^<0,001; ^g^0,017; ^†^0,027; ^††^0,001; ^†††^0,04, ^††††^0,001; ^†††††^<0,001, ^††††††^<0,001; ^†††††††^0,017.

### Detection of CTCs before the initiation of systemic treatment using immunofluorescence

**Figure 1:**
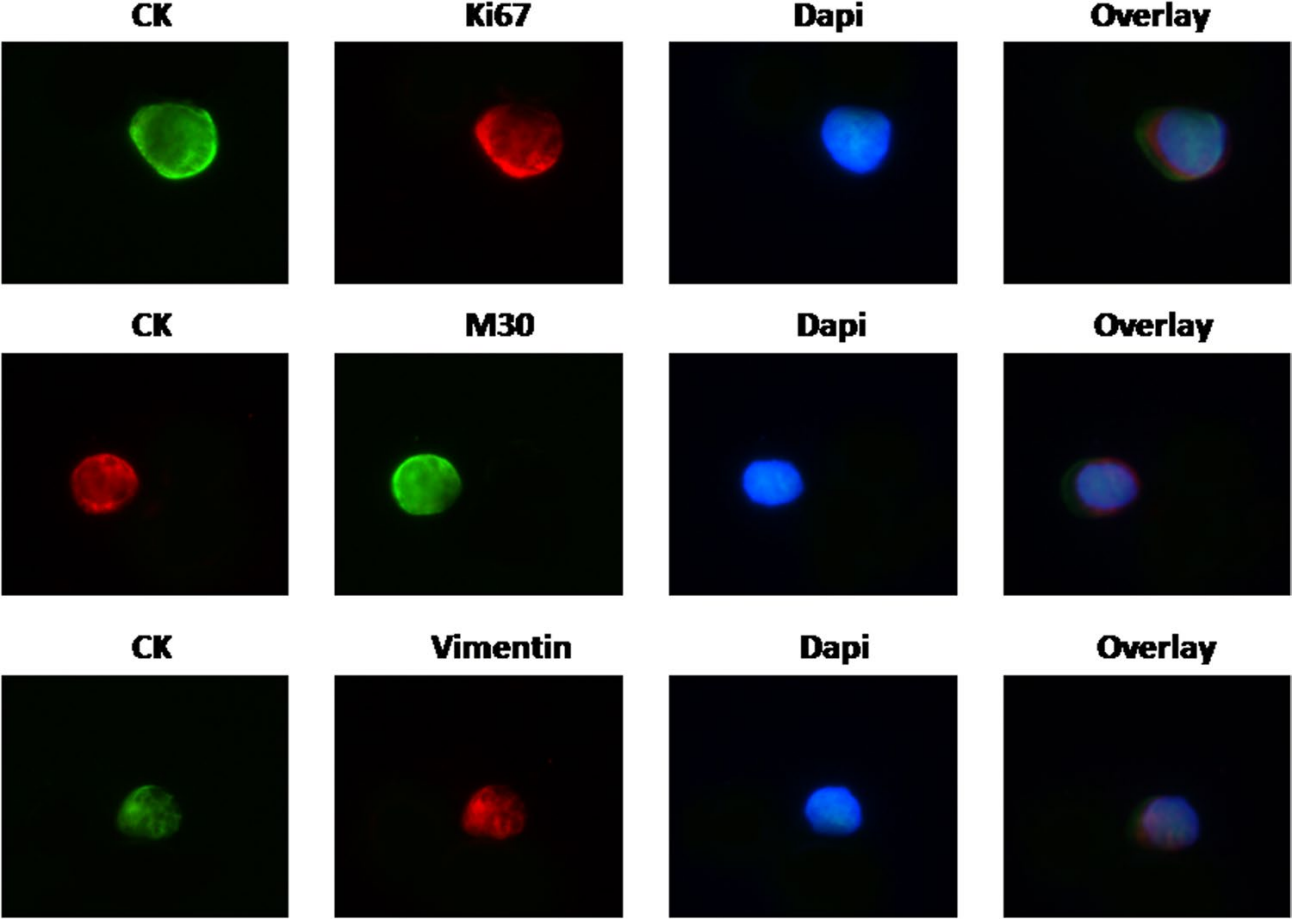
CK^+^/Ki67^+^ (**a**), CK^+^/M30^+^ (**b**) and CK^+^/Vim^+^ (**c**) CTCs by double immunofluorescense staining.

Patients’ CTCs were further characterized using antibodies against the Ki67, M30 and Vimentin (Vim). Figure 1 indicates representative patients’ CTCs stained with the above mentioned antibodies. Table 2 demonstrates that at baseline, 31 (53.4%) and 39 (67.2%) patients had detectable proliferative (CK^+^/Ki67^+^) and non-proliferative (CK^+^/Ki67^−^) CTCs, respectively. The detection of CTCs with a proliferative phenotype was not related to the detection of CTCs using the CS platform (*p* = 0.108) (Table 3). In addition, most patients (72.5%) had non-apoptotic (CK^+^/M30^−^) CTCs and this cell population was mainly observed in patients with an increased number of CTCs as assessed by CS (Tables 2 and 3; *p* = 0.022); conversely, only a small number (15.5%) of patients had apoptotic (CK^+^/M30^+^) CTCs at baseline (Table 2). Moreover, CTCs were mainly CK^+^/Vim^+^ in 43 (74.1%) patients, suggesting that these cells undergo EMT; however, 56.9% of the tested patients had also detectable CK^+^/Vim^−^ CTCs (Table 2). Both populations of CK^+^/Vim^+^ and CK^+^/Vim^−^ CTCs were associated with the number of CTCs detected by CS (*p* = 0.014 and *p* = 0.001, respectively) (Table 3).

**Table 3:** Heterogeneity of CTC sub-populations according to Cellsearch positivity before treatment with pazopanib.

Phenotype	CellSearch platform (N = 56)		p value
	≥5 CTCs (N = 28)	<5 CTCs (N = 28)	
CK^+^/Ki67^+^	12 (42,8%)	18 (64,3%)	0,108
CK^+^/Ki67^−^	23 (82,1%)	16 (57,1%)	0,042
CK^+^/M30^+^	7 (25,0%)	1 (3,6%)	0,131
CK^+^/M30^−^	23 (82,1%)	18 (64,3%)	0,022
CK^+^/Vim^+^	25 (89,3%)	17 (60,7%)	0,014
CK^+^/Vim^−^	22 (78,6%)	10 (35,7%)	0,001

Αmong the 12 (21.4%) patients without detectable CTCs by CS (0 CTCs/7.5 ml of blood), 8 (66.7%), 1 (8.3%) and 7 (58.3%) had CK^+^/Ki67^+^, CK^+^/M30^+^ and CK^+^/Vim^+^ CTCs, respectively (Supplementary Table 1). It is to note that in 8 out of 12 patients with detectable CTCs by IF but not by CS, all the CTCs’ subpopulations with proliferative, apoptotic and EMT phenotypes were present (Supplementary Table 2); however, double IF revealed that no one of these patients had detectable EpCAM^+^ CTCs. Furthermore, it is to note that no CK^+^/EpCAM^+^ or Vim^+^/EpCAM^+^ CTCs could be detected in the remaining 4 patients without detectable CTCs by CS (Supplementary Table 2).

### Effect of Pazopanib on CTCs

**Figure 2:**
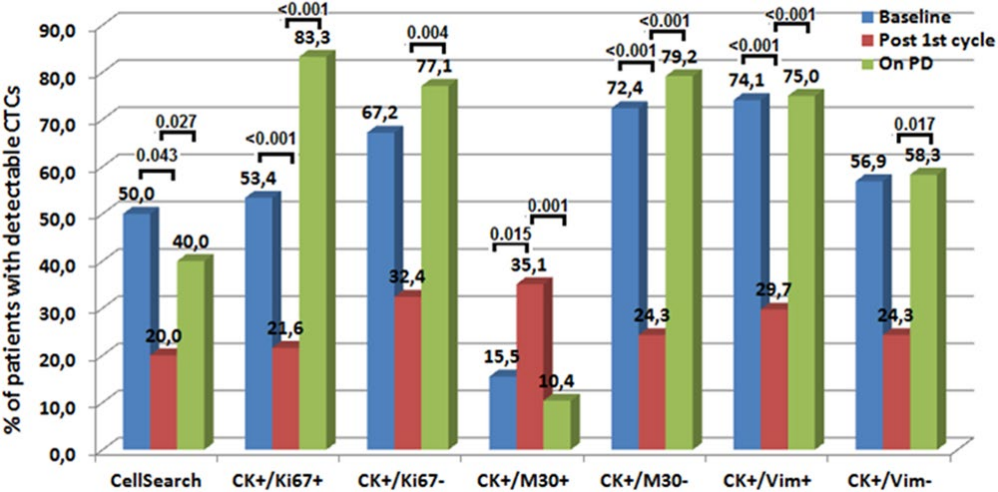
Percentage of SCLC patients with detectable circulating tumor cells (CTCs) at baseline, after one-cycle of treatment and at the time of disease progression (PD).

The administration of one pazopanib cycle resulted in a significant decrease of the patients with a high CTC number as well as of the absolute number of CTCs compared to pre-treatment values (Fig. 2 and Table 2; *p* = 0.043). Similarly, one cycle of pazopanib resulted in: (i) a significant decrease of the number of patients with proliferative (CK^+^/Ki67^+^) (21.6%; *p* < 0.001) but not of non-proliferative (CK^+^/Ki67^−^) CTCs; (ii) a significant increase of patients with apoptotic (CK^+^/M30^+^) CTCs (35.1%; *p* = 0.015) and in a significant decrease of the non-apoptotic (CK^+^/M30^−^) CTCs (24.3%; *p* < 0.001) and (iii) a significant decrease of the number of patients with CK^+^/Vim^+^ CTCs (29.7%; *p* < 0.001) but not of CK^+^/Vim^-^ CTCs (Fig. 2, Supplementary Fig. 1 and Table 2).

### Detection of CTCs at the time of disease progression

At the time of disease progression, 18 (40.0%) out of 45 evaluable patients had an increased number of CTCs (median: 73 CTCs/7.5 ml; range, 0–16806) (Fig. 2, Supplementary Fig. 1 and Table 2). The phenotypic characterization of the CTCs revealed that both the incidence of detection and the median number of the different CTC sub-populations were significantly increased compared to post-1^st^ cycle values (Supplementary Fig. 1 and Table 2). The only exception from this observation concerned the incidence of detection and the median number of apoptotic (CK^+^/M30^+^) CTCs, which was significantly decreased at the time of disease progression (Supplementary Fig. 1 and Table 2).

### Detection of CTCs and clinical outcome

An objective response (PR; Partial Response) was achieved in seven (12.5%) patients while 19 (33.9%) and 30 (53.6%) patients, experienced stable (SD) and progressive disease (PD), respectively. Patients with disease progression (PD) had a significantly higher number of detectable CTCs at baseline as assessed by CS compared to patients with PR or SD (PD median number of 17 CTCs/7.5 ml; PR median number of 0 CTCs/7.5 ml and SD median number of 2 CTCs/7.5 ml, respectively; *p* = 0.006, Supplementary Table 3). Nevertheless, clinical response to pazopanib was not associated with the presence of any subpopulation of CTCs (Supplementary Table 3). The median PFS and OS for all patients was 2.7 months (95% CI: 2.1–3.3) and 6.5 months (95% CI: 4.9–8.2), respectively. There was no difference of PFS or PS according to the presence of the different CTC sub-populations except in the case of patients with detectable CK^+^/Vim^+^ CTCs after one-cycle of pazopanib who had a significantly shorter OS compared to patients with CK^+^/Vim^−^ CTCs (Supplementary Fig. 2). In addition, patients with a high number of CTCs by CS at baseline had significantly shorter PFS and OS than patients with <5 CTCs/7.5 ml of blood [PFS: 1.9 months (95% CI: 0.9–2.8) vs 3.6 months (95% CI: 1.3–5.9); *p* < 0.001 and OS: 5.2 months (95% CI: 2.4–7.9) vs 10.1 months (95% CI: 7.4–12.8) (*p* = 0.001)] (Supplementary Fig. 3).

### Univariate and Multivariate Analysis

Univariate analysis revealed that lactate dehydrogenase (LDH), anaemia, decreased sensitivity to platinum and the detection of a high number of CTCs by CS at baseline CTC were significantly associated with a shorter PFS (Table 4). In multivariate analysis adjusting for these factors, only sensitivity to platinum and the detection of a high number of CTCs emerged as independent factors associated with reduced PFS (HR: 3.0, *p* = 0.001 and HR:4.9; *p* < 0.001, respectively) (Table 4).

**Table 4:** Univariate and multivariate Cox regression analysis.

	Univariate Analysis				Multivariate Analysis			
	PFS		OS		PFS		OS	
	HR (95, 0%CI)	Sig.	HR (95, 0%CI)	Sig.	HR (95, 0%CI)	Sig.	HR (95, 0%CI)	Sig.
LDH (High vs Normal)	2,0 (1,1–3,6)	0,028	0,5 (0,–0,9)	0,016	—	—	4,7 (1,7–13,0)	0,003
Hct (Low vs Normal)	2,1 (1,1–4,1)	0,024	2,0 (1,0–3,8)	0,043	—	—	—	—
Group (Resistant vs Sensitive)	3,2 (1,7–6,2)	<0,001	2,5 (1,4–4,6)	0,003	3,0 (2,0–6,0)	0,001	2,0 (1,0–5,0)	0,001
CTCs (CS) at baseline (≥5 vs <5 CTCs)	3,4 (1,8–6,6)	<0,001	2,9 (1,5–5,4)	0,001	4,9 (2,3–10,6)	<0, 001	—	—
CTCs (CS) post-1st cycle (≥5 vs <5 CTCs)	1,3 (0,6–3,2)	0,486	2,7 (1,1–6,7)	0,034	—	—	—	—
CTCs (CS) on PD (≥5 vs <5 CTCs)	—	—	2,3 (1,2–4,3)	0,011	—	—	2,0 (1,0–6,0)	0,005
CK^+^/Ki67^+^ vs CK^+^/Ki67^−^ CTCs (baseline)	1,1 (0,6–2,0)	0,722	0,8 (0,5–1,5)	0,51	—	—	—	—
CK^+^/Ki67^+^ vs CK^+^/Ki67^−^ CTCs (post 1st cycle)	1,0 (0,5–2,3)	0,949	1,3 (0,6–2,9)	0,538	—	—	—	—
CK^+^/Ki67^+^ vs CK^+^/Ki67^−^ (PD)	—	—	1,1 (0,5–2,3)	0,909	—	—	—	—
CK^+^/M30^+^ vs CK^+^/M30^−^ (baseline)	1,2 (0,5–2,8)	0,735	1,6 (0,6–3,7)	0,326	—	—	—	—
CK^+^/M30^+^ vs CK^+^/M30^−^ (post 1st cycle)	1,5 (0,8–3,1)	0,283	1,3 (0,6–2,6)	0,537	—	—	—	—
CK^+^/M30^+^ vs CK^+^/M30^−^ (PD)	—	—	1,9 (0,6–5,4)	0,249	—	—	—	—
CK^+^/Vim^+^ vs CK^+^/Vim^−^ (baseline)	1,6 (0,8–3,2)	0,200	1,6 (0,8–3,2)	0,224	—	—	—	—
CK^+^/Vim^+^ vs CK^+^/Vim^−^ (post 1st cycle)	1,9 (0,8–4,1)	0,126	4,9 (2,1–11,8)	<0,001	—	—	7,9 (2,9–21,8)	<0,001
CK^+^/Vim^+^ vs CK^+^/Vim^−^ (PD)	—	—	1,3 (0,7–2,5)	0,455	—	—	—	—

Similarly, univariate analysis indicated that LDH, anaemia, sensitivity to platinum, the detection of a high number of CTCs by CS at all tested time points and the detection of CK^+^/Vim^+^ CTCs, were significantly associated with a better OS (Table 4). In multivariate analysis adjusting for these factors, LDH, decreased sensitivity to platinum, increased number of CTCs on disease progression and detection of CK^+^/Vim^+^ CTCs after one treatment cycle emerged as independent prognostic factors associated with a decreased OS (HR: 4.7, *p* = 0.003; HR: 2.0, *p* = 0.001; HR: 2.0, *p* = 0.005 and HR: 7.9, *p* < 0.001, respectively) (Table 4).

## Discussion

To our knowledge this is the first study evaluating the dynamic role of different subpopulations of CTCs in patients with SCLC treated with an anti-angiogenic agent (pazopanib) in the context of a phase II study which enrolled patients with recurrent and resistant/refractory disease. The rational for this study was based on previous data from our group indicating that CTCs from patients with early and/or metastatic breast cancer co-express HIF-1 as well as both VEGF and VEGR2, suggesting that some CTCs, which are hypoxic, might have activated an autocrine angiogenesis pathway by expressing VEGFR2 on their membranes and producing VEGF^22^. The results of the current study clearly indicate that the administration of even one pazopanib cycle resulted in a significant decrease of patients with an increased CTC number (>5CTCs/7.5 mL of blood). Moreover, the absolute number of CTCs after one cycle of pazopanib was significantly decreased compared with the pre-treatment values. Conversely, at the time of disease progression both the number of patients with an increased number of CTCs and the median number of CTCs were significantly increased at their baseline levels. These findings are in agreement with previous studies supporting the dynamic value of the changes of CTCs after one treatment cycle in SCLC patients receiving front line treatment^13,37,38^.

The current study also confirmed the phenotypic heterogeneity of CTCs in patients with SCLC as already reported by Hou *et al*.^13^ Indeed, double immunofluorescent staining of CTCs demonstrated that both proliferative (CK^+^/Ki67^+^) and non-proliferative (CK^+^/Ki67^−^), apoptotic (CK^+^/M30^+^) and non-apoptotic (CK^+^/M30^−^) as well as CK^+^/Vim^+^ and CK^+^/Vim^−^ CTCs were present at the same time in patients before the initiation of any systemic treatment. In addition, the presented data demonstrate that both the incidence of detection and the median number of different CTC sub-populations are associated with the treatment effect. Indeed, both the incidence of detection as well as the median number of proliferative and CK^+^/Vim^+^ CTCs were mainly detected before the administration of pazopanib, whereas after one treatment cycle, the incidence of their detection and their median number were significantly decreased; conversely, at the time of disease progression, both sub-populations of CTCs were significantly increased. On the other hand, one cycle of pazopanib was associated with an increased number of apoptotic (CK^+^/M30^+^) and a decreased number of non-apoptotic (CK^+^/M30^−^) CTCs whereas the opposite was observed at the time of disease progression. These findings further support the pharmacodynamic value of the different sub-populations of CTCs, suggesting that the evaluation of the proliferating and apoptotic CTCs during treatment might provide a useful biomarker to monitor treatment efficacy in patients with SCLC. Nevertheless, we have to be cautious since these findings were obtained in patients with relapsed disease treated with an anti-angiogenic agent, which does not represent the standard of care for these patients. Therefore, this hypothesis should be validated in a future prospective study in newly diagnosed SCLC patients treated with standard chemo-radiotherapy or chemotherapy. Currently, Hughes *et al*., developed a novel platform to rapidly screen drug efficacies of chemotherapeutics, using CTC enumeration as a diagnostic output and predictor for drug susceptibility in cancer patients^39^. Since, the assay they developed is intended to be performed *in vivo* rather than *in vitro*, our study might also provide a necessary step towards the use of CTCs as a predictive clinical tool.

The current study also demonstrated that treatment efficacy, as defined by disease control rate at 8 weeks, was significantly associated with the number of detectable CTCs by CS, but not by immunofluorescence (Supplementary Table 3). Moreover, the detection of an increased number of CTCs by CS is associated with a decreased PFS and OS, and multivariate analysis revealed that this was emerged as an independent prognostic factor (Table 4); this observation is in agreement with previous reports irrespectively of the used method for the detection of CTCs^13,38,40–45^. An interesting finding in the current study is the observation that the presence of CK^+^/Vim^+^ CTCs after one cycle of pazopanib was associated with a dismal clinical outcome and was emerged as an independent factor associated with decreased OS (Supplementary Fig. 2). This observation further supports that the phenotypic monitoring of the CK^+^/Vim^+^ CTCs might be used as a dynamic prognostic biomarker. In patients without detectable CTCs by CS, double immunofluorescence staining revealed the presence of both CK^+^/Ki67^+^ and CK^+^/Vim^+^ CTCs whilst the detection of apoptotic cells was rare (Supplementary Table 1). It is well established that CTCs undergo EMT and that the percentage of CTCs undergoing EMT may vary between patients. The CK^+^/Vim^+^ CTCs are considered to represent tumor cells undergoing EMT during their hematogenous dissemination^46–48^. CTCs undergoing EMT have been reported to weakly express epithelial surface antigens, like cytokeratins and EpCAM^40^. Since the CS platform is based on the recognition and capture of EpCAM^+^ CTCs it is obvious that CTCs undergoing EMT are less likely to be detected by this system. It is noteworthy that in this small group of patients with undetectable CTCs by CS, most patients displayed EpCAM-negative proliferating and not apoptotic CTCs. (Supplementary Table 2) This observation seems to indicate that some subpopulations of CTCs (i.e. proliferating EpCAM^−^ or EpCAM^−^ CTCs undergoing EMT) may be of particular clinical relevance representing, more appropriately the biologic behavior of the tumor. Further studies are required in order to elucidate this hypothesis.

In conclusion, the data presented in the current study clearly demonstrate that the enumeration and the phenotypic characterization of CTCs in patients with SCLC treated with pazopanib are of clinical relevance with predictive and prognostic value. In addition, the analysis of the different subpopulations of CTCs during this anti-angiogenic treatment could be considered as a dynamic biomarker for the monitoring of treatment efficacy. Subsequent studies in larger prospective cohorts have to evaluate whether these phenotypic changes of CTCs during standard front-line treatment could validate their use as a valuable real time biomarker in patients with SCLC.

## Electronic supplementary material

Dataset 1
