# Supplementary material for: Dynamic changes of phenotypically different circulating tumor cells sub-populations in patients with recurrent/refractory small cell lung cancer treated with pazopanib
Source: Sci Rep. 2018 Feb 2;8:2238. doi: 10.1038/s41598-018-20502-1 (PMC5797076; doi:10.1038/s41598-018-20502-1)
Supplement: Supplementary file 1 — Dataset 1 [file 41598_2018_20502_MOESM1_ESM.doc]

**Dynamic changes of phenotypically different circulating tumor cells sub-populations in patients with recurrent/refractory small cell lung cancer treated with pazopanib**

Ippokratis Messaritakis^1^, Eleni Politaki^1^, Fillipos Koinis^1^, Dimitris Stoltidis^2^, Stella Apostolaki^1^, Maria Plataki^1^, Eleftheria-Kleio Dermitzaki^2^, Vassilis Georgoulias^1,2^, Athanasios Kotsakis^1,2^

^1^Laboratory of Tumor Cell Biology, Medical School, University of Crete, Heraklion, Crete, Greece

^2^Department of Medical Oncology, University General Hospital of Heraklion, Crete, Greece

**Supplementary Table S1:** Detection of different phenotypes of CTCs in patients with <5 CTCs/7.5ml of blood by CellSearch

| **CTCs /7,5ml of blood (CellSearch)** | **CK^+^Ki67^+^ CTCs/10^6^ PBMCs** | **CK^+^M30^+^ CTCs/10^6^ PBMCs** | **CK^+^Vim^+^ CTCs/10^6^ PBMCs** |
| --- | --- | --- | --- |
| **<5 (n=28)** | 18 (64,3%) | 1 (3,6%) | 17 (60,7%) |
| **1-4 (n=16)** | 10 (62,5%) | 0 (0,0) | 10 (62,5%) |
| **0 (n=12)** | 8 (66,7%) | 1 (8,3%) | 7 (58,3%) |

**Suppl. Table S2:** Detection of CTCs subpopulations with immunofluorescence in patients without detectable CTCs by CS

| **Patient's No** | **CK+/EpCam+** | **Vim+/EpCam+** | **CK+/Ki67+** | **CK+/Ki67-** | **CK+/M30+** | **CK+/M30-** | **CK+/VIM+** | **CK+/VIM-** |
| --- | --- | --- | --- | --- | --- | --- | --- | --- |
| **1** | 0 | 0 | 1 | 2 | 0 | 2 | 0 | 2 |
| **2** | 0 | 0 | 6 | 0 | 0 | 2 | 13 | 1 |
| **3** | 0 | 0 | 8 | 1 | 0 | 7 | 13 | 2 |
| **4** | 0 | 0 | 7 | 1 | 0 | 10 | 12 | 4 |
| **5** | 0 | 0 | 9 | 2 | 0 | 7 | 12 | 7 |
| **6** | 0 | 0 | 2 | 5 | 0 | 4 | 2 | 0 |
| **7** | 0 | 0 | 8 | 2 | 0 | 4 | 4 | 1 |
| **8** | 0 | 0 | 8 | 1 | 1 | 9 | 12 | 1 |
| **9** | 0 | 0 | 0 | 0 | 0 | 0 | 0 | 0 |
| **10** | 0 | 0 | 0 | 0 | 0 | 0 | 0 | 0 |
| **11** | 0 | 0 | 0 | 0 | 0 | 0 | 0 | 0 |
| **12** | 0 | 0 | 0 | 0 | 0 | 0 | 0 | 0 |
|  |  |  |  |  |  |  |  |  |

**Suppl. Table S3:** Objective responses to pazopanib according to the phenotype of CTCs at baseline

|  | **CellSearch (N=56)** | | | | | **IF** | | | | | | | | |
| --- | --- | --- | --- | --- | --- | --- | --- | --- | --- | --- | --- | --- | --- | --- |
|  | **All patients** | **≥5 CTC (%) (N= 28)** | **<5 CTCs (%) (N= 28)** | **Median (Range)** | **p** | **CK^+^/**  **Ki67^+^** | **CK^+^/**  **Ki67^-^** | ***p*** | **CK^+^/**  **M30^+^** | **CK^+^/**  **M30^-^** | ***p*** | **CK^+^/**  **Vim^+^** | **CK^+^/**  **Vim^-^** | ***p*** |
| **PR** | 7(12,5%) | 2(3,6%) | 5(8,9%) | 0(0-388) | 0,006 | 4(6,9%) | 4(6,9%) | 0,891 | 2(3,4%) | 6(10,3%) | 0,298 | 6(10,3%) | 2(3,4%) | 0,541 |
| **SD** | 19(33,9%) | 5(8,9%) | 14(25,0%) | 2(0-27) |  | 11(19,0%) | 8(13,8%) |  | 1(1,7%) | 18(31,0%) |  | 12(20,7%) | 7(12,1%) |  |
| **PD** | 30(53,6%) | 21(37,5%) | 9(16,1%) | 17(0-11143) |  | 16(27,6%) | 15(25,9%) |  | 6(10,3%) | 25(43,1%) |  | 24(41,4%) | 7(12,1%) |  |

**Supplementary Fig 1**: Spaghetti plots showing how CTC numbers, expressing different phenotypes, change during treatment with pazopanib


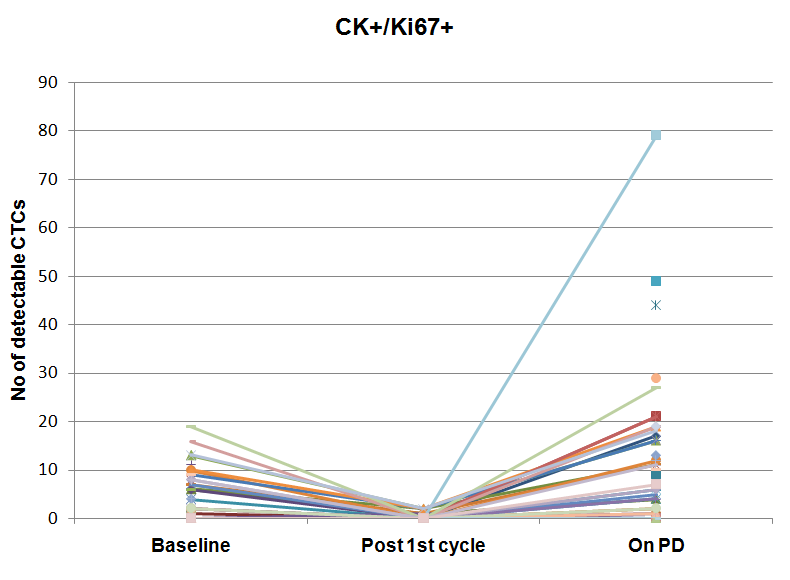

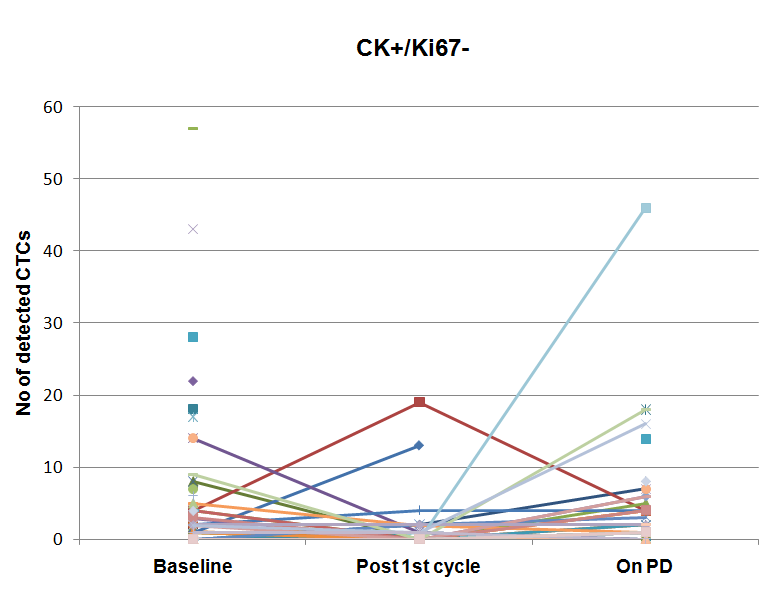


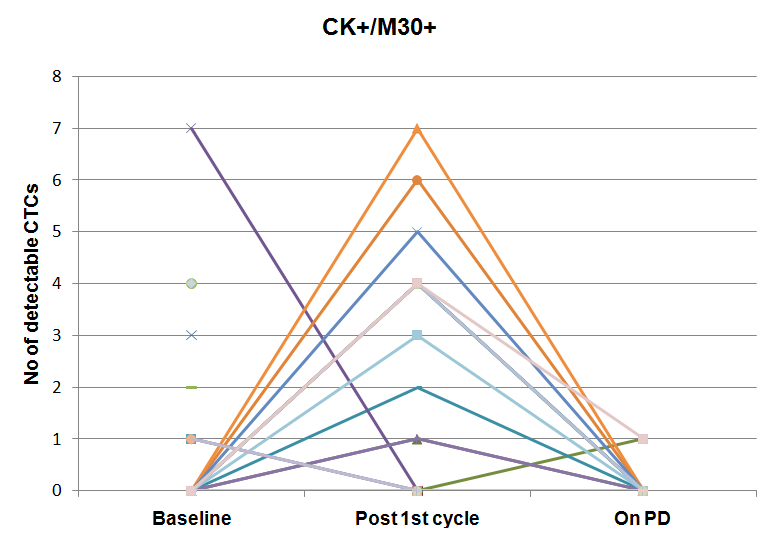

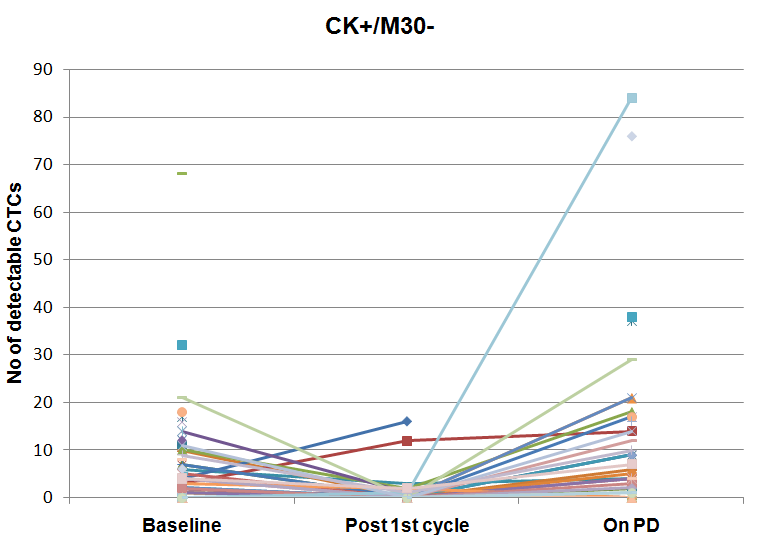


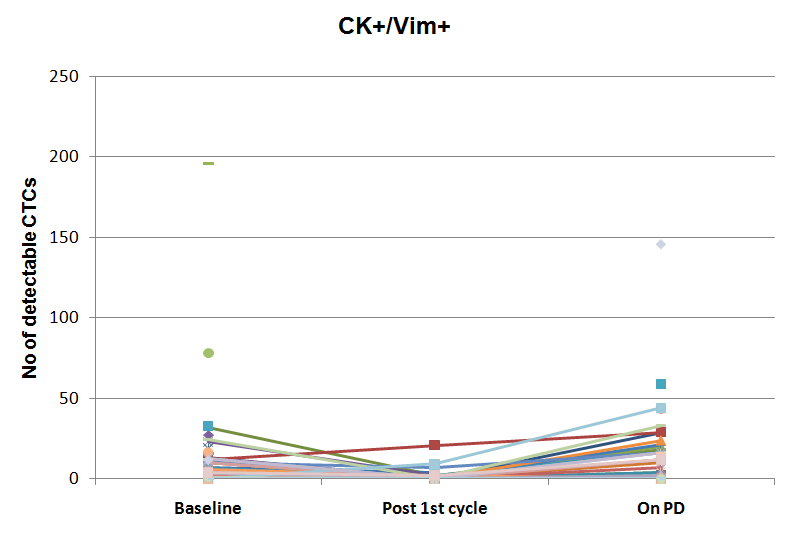

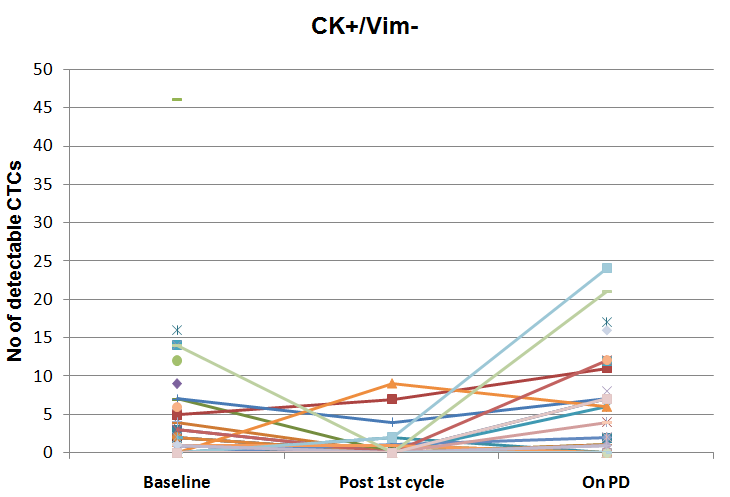


**Supplementary Fig 2:** Kaplan-Meier curve for OS after one treatment cycle, according to the detection of CK+/Vim+ CTCs


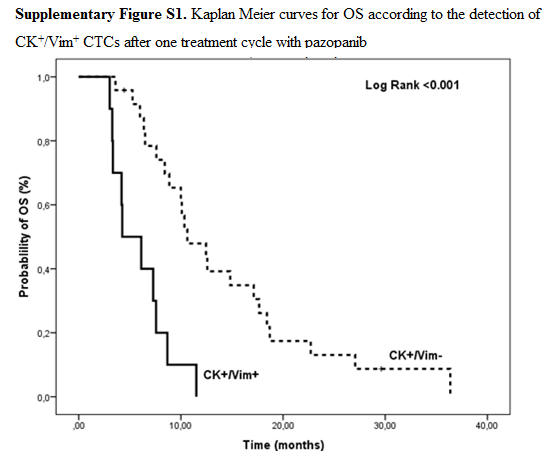


**Supplementary Fig 3:** Kaplan-Meier curves for PFS (a) and OS (b) at baseline, according to the detection of CTCs by the CellSearch

**
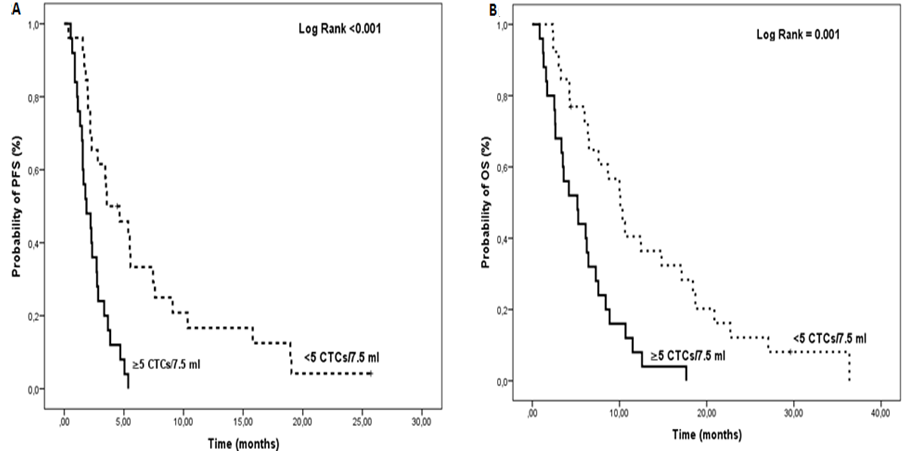
**
